# Chikungunya virus nsP1 interacts directly with nsP2 and modulates its ATPase activity

**DOI:** 10.1038/s41598-018-19295-0

**Published:** 2018-01-18

**Authors:** Sameer Kumar, Abhishek Kumar, Prabhudutta Mamidi, Atul Tiwari, Sriram Kumar, Animamalar Mayavannan, Sagarika Mudulli, Ajit Kumar Singh, Bharat Bhusan Subudhi, Soma Chattopadhyay

**Affiliations:** 1 Institute of Life Sciences, Bhubaneswar, India; 2 Banaras Hindu University, Varanasi, U.P. India; 3 Anna University, Chennai, India; 4 School of Pharmaceutical Sciences, Siksha O Anusandhan University, Bhubaneswar, India

## Abstract

Chikungunya virus (CHIKV) is a mosquito-borne virus, which has created an alarming threat in the world due to unavailability of vaccine and antiviral compounds. The CHIKV nsP2 contains ATPase, RTPase, helicase and protease activities, whereas, nsP1 is a viral capping enzyme. In alphaviruses, the four non-structural proteins form the replication complex in the cytoplasm and this study characterizes the interaction between CHIKV nsP1 and nsP2. It was observed that, both the proteins co-localize in the cytoplasm and interact in the CHIKV infected cells by confocal microscopy and immunoprecipitation assay. Further, it was demonstrated through mutational analysis that, the amino acids 1-95 of nsP2 and 170-288 of nsP1 are responsible for their direct interaction. Additionally, it was noticed that, the ATPase activity of nsP2 is enhanced in the presence of nsP1, indicating the functional significance of this interaction. *In silico* analysis showed close (≤1.7 Å) polar interaction (hydrogen bond) between Glu^4^, Arg^7, 96, 225^ of nsP2 with Lys^256, 206^, Val^367^ and Phe^312^ of nsP1 respectively. Hence, this investigation provides molecular characterization of CHIKV nsP1-nsP2 interaction which might be a useful target for rational designing of antiviral drugs.

## Introduction

In the year 1955, Marion Robinson and W.H.R. Lumsden described a viral outbreak in 1952 on the Makonde Plateau and Tanganyika region, which they named as Chikungunya fever (CHIKF)^1,2^. Chikungunya virus (CHIKV) is the causative agent of CHIKF and transmitted by *Aedes sp*. mosquitoes^3^. CHIKF is an acute infection and may lead to chronic Chikungunya virus-induced arthralgia^4,5^. The symptoms which predominantly discriminate it from other mosquito-borne infections (like dengue, malaria and Zika fever) are, high grade fever (108 °F), arthralgia (joint pain) and maculopapular rash^4,6,7^. In 2005, an outbreak in the Reunion island caused CHIKV infection in one-third of its population^8^ and in 2006, nearly 1.25 million people were affected in India^9^. Most recently, in the year 2013-14 there were more than one million individuals infected by this virus throughout the America^10^.

CHIKV is a small (60–70 nm in diameter), positive sense, single-stranded RNA virus which belongs to the Togaviridae family of alphavirus genus^11^. The genome is around 11.8 kb long and encodes four nonstructural proteins (nsPs) (nsP1, nsP2, nsP3 and nsP4) which help in the synthesis of the viral RNA. The genome also encodes for three main structural proteins (Capsid, E1 and E2) which form the hetero-dimeric spikes on the virion’s surface and two small cleavage products (E3 and 6K)^6,12^.

CHIKV is closely related to Semliki Forest virus (SFV) and Sindbis virus, hence most of the functions of nsPs were predicted in comparison to these alphaviruses^11^. The CHIKV nsP1 is a 535 amino acid (aa) long protein. According to the various computational analysis of homologous structural and functional prediction, three domains have been described for nsP1^13^. These are the N-terminus (NT) methyltransferase (MT) and guanylyltransferase domain (1-170 aa), the middle membrane binding (MB) domain (171-300 aa) and the C-terminus (CT) D3 domain (301-535 aa)^13^. The NT domain is involved in methylation and capping of the newly synthesized RNA in its 5′ region^14–16^. The middle domain anchors the replication complex (RC) with the cellular membranes^17^ with the help of palmitoylation in the cysteine residues (418-420 aa in SFV)^18–20^. The function of nsP1-CT is not well characterized yet for any of the alphaviruses. Two major functional domains for CHIKV nsP2 (798 aa) are characterized which are the NT (1-456 aa) and the CT domain (457-798 aa)^13^. The nsP2 protein is multifunctional which contains the helicase, nucleoside triphosphatase (NTPs) and RNA-triphosphatase (RTPase) activities in its NT while the nsP2-CT contains protease activity^21–23^. The CHIKV nsP3 possesses ADP-ribose-1-phosphate phosphatase and RNA-binding activities^24^. The nsP4 is an RNA-dependent RNA-polymerase (RdRp)^25,26^ protein which also plays a role in scaffolding the interaction with other nsPs and host proteins through its NT domain^27^.

During alphavirus replication, the nsPs are first translated as a polyprotein precursor (P1234), where nsP2 helps in cleavage and processing of mature nsP4^28,29^. Then, the mature nsP4 protein with P123 polyprotein forms the early RC and transcribes the negative sense RNA^30,31^. During the late phase of replication, nsP2 cleaves and processes all the mature nsPs, which form the late RC to synthesize the positive sense 46S genomic and 26S subgenomic RNA (the precursor RNA for structural proteins)^30,31^. There are several reports in alphaviruses, that nsPs interact with each other in the RC for smooth operation of the replication machinery^27,32–34^. Besides, it has also been shown that the alphavirus nsP3 and nsP4 with nsP1 helps in the synthesis of minus-strand RNA genome in RC^31,35^.

Earlier, it was reported that the CHIKV nsP2 protein interacts with CHIKV nsP1 in yeast two-hybrid (Y2H), ELISA and GST pull-down assay^13,36^. However, the detailed characterization of this interaction was obscured during infection. Accordingly, in this investigation, the interaction of nsP1-nsP2 has been demonstrated during CHIKV infection in Vero cells, the interacting domains have been mapped and the functional significance of this interaction has been explored *in vitro*.

## Results

### CHIKV nsP2 interacts with nsP1 during virus infection in Vero cells through RC

**Figure 1:**
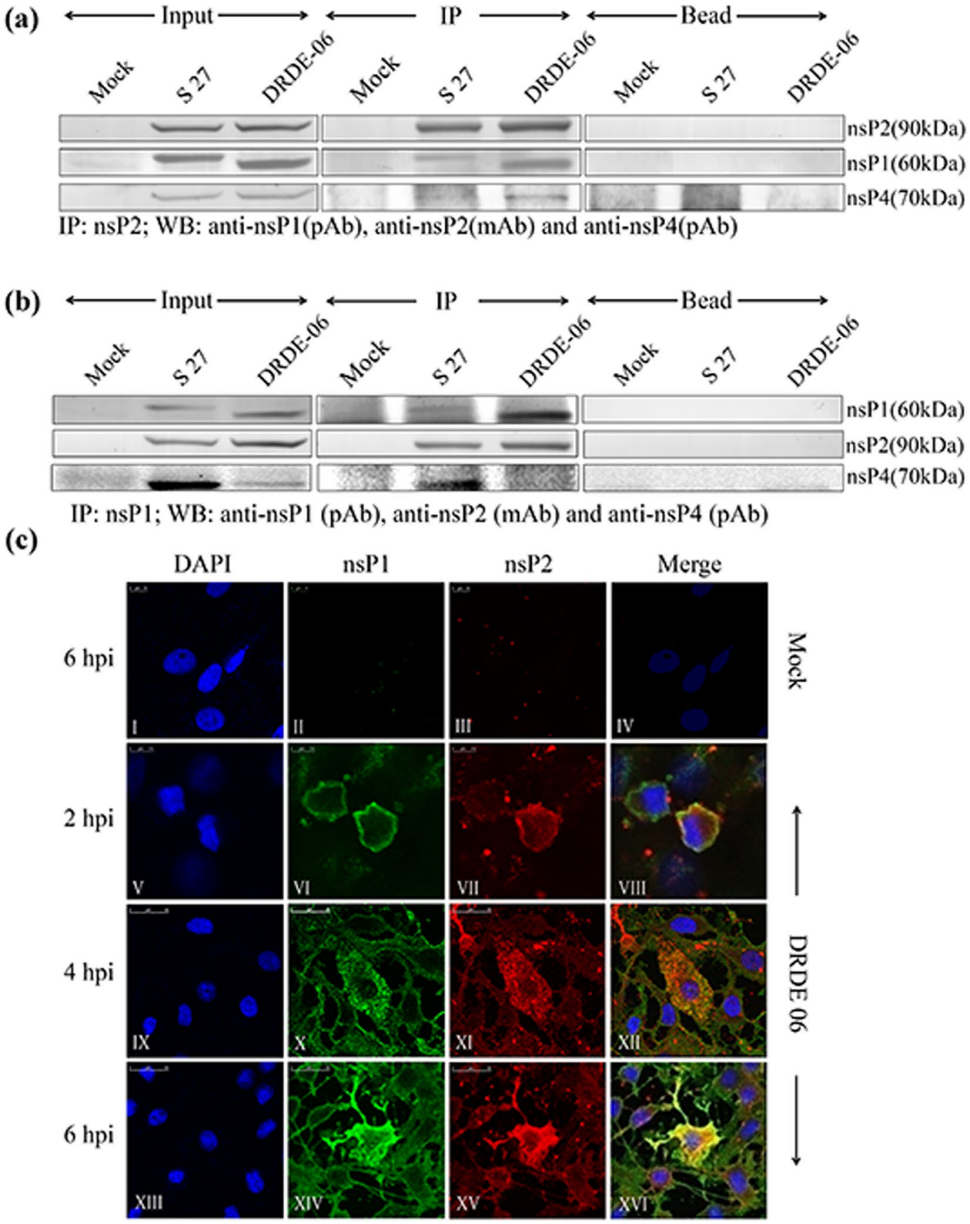
CHIKV nsP2 interacts with nsP1 during virus infection in Vero cells through RC. (**a**) Vero cells grown in 100 mm dish, were either infected with S 27 or DRDE-06 virus at an MOI of 2. Mock infected cells were considered as negative control. The cells were harvested at 6 hpi and 10 hpi for DRDE-06 and S 27 respectively. Immunoprecipitation was performed with the lysates using CHIKV anti-nsP2 mAb and Western blot was carried out using anti-nsP2 mAb, anti-nsP1 and anti-nsP4 pAbs. Beads with normal IgG serve as negative control. (**b**) Same Vero cell lysates were immunoprecipitated with anti-nsP1 pAb and elutes were separated in 10% SDS-PAGE. Western blot was performed using anti-nsP1 pAb, -nsP2 mAb and -nsP4 pAb. (**c**) Vero cells were plated onto cover-slips and infected either without virus (mock) or with DRDE-06 at a MOI of 2. The cells were fixed after 2, 4 and 6 hpi, probed together with anti-nsP1 pAb (II, VI, X, XIV) and anti-nsP2 mAb (III, VII, XI, XV) followed by staining with secondary antibodies, anti-rabbit Alexa Fluor 488 (green) or anti-mouse Alexa Fluor 594 (red) respectively. Nuclei were counterstained with DAPI (blue). Fluorescent images were acquired using the Leica TCS SP5 confocal microscope.

In the present investigation, immunoprecipitation (IP) was performed to understand the interaction of nsPs during CHIKV infection in Vero cells. In addition to the prototype strain of CHIKV (S 27), a 2006 Indian outbreak strain, DRDE-06 was also included in this study. Our earlier observation demonstrated that DRDE-06 exhibits faster replication than S 27 in mammalian cells^37^, hence, the CHIKV infection was carried out and the cells were harvested at 10 hour post infection (hpi) for S 27 and 6 hpi for DRDE-06. The CHIKV nsP2 or nsP1 protein was immunoprecipitated from infected Vero cell lysates using the anti-nsP2 monoclonal antibody (mAb) or anti-nsP1 polyclonal antibody (pAb) respectively. The protein complex was resolved in 10% SDS-PAGE and Western blot analysis was conducted. As shown in Fig. 1a and b, it was observed that the nsP2 protein interacts with nsP1 during CHIKV infection and vice versa. It was also observed that the nsP4 protein was present in the nsP1-nsP2 complex (Fig. 1a and b); however, neither nsP3 nor E2 was detected in the Western blot (data not shown). To monitor the colocalization of CHIKV nsP1 and nsP2, Vero cells were infected with DRDE-06 at MOI 2 and cells were fixed at three different time points (2, 4, 6 hpi). It was demonstrated that at 2 hpi the CHIKV nsP1 protein (green fluorescence) was mostly present in the plasma membrane and co-localized with nsP2 (red fluorescence) (Fig. 1c VIII). At 4 and 6 hpi, the CHIKV nsP1 protein co-localized with nsP2 in the cytoplasmic compartment of the infected cells (Fig. 1c XII and XVI). Together, the results suggest that, CHIKV nsP2 and nsP1 interacts with each other and co-localizes during infection in mammalian cells through the formation of RC. The data also indicate that, nsP4 interacts with nsP2 and nsP1 during infection.

### CHIKV nsP2-NT directly interacts with nsP1-WT *in vitro*

**Figure 2:**
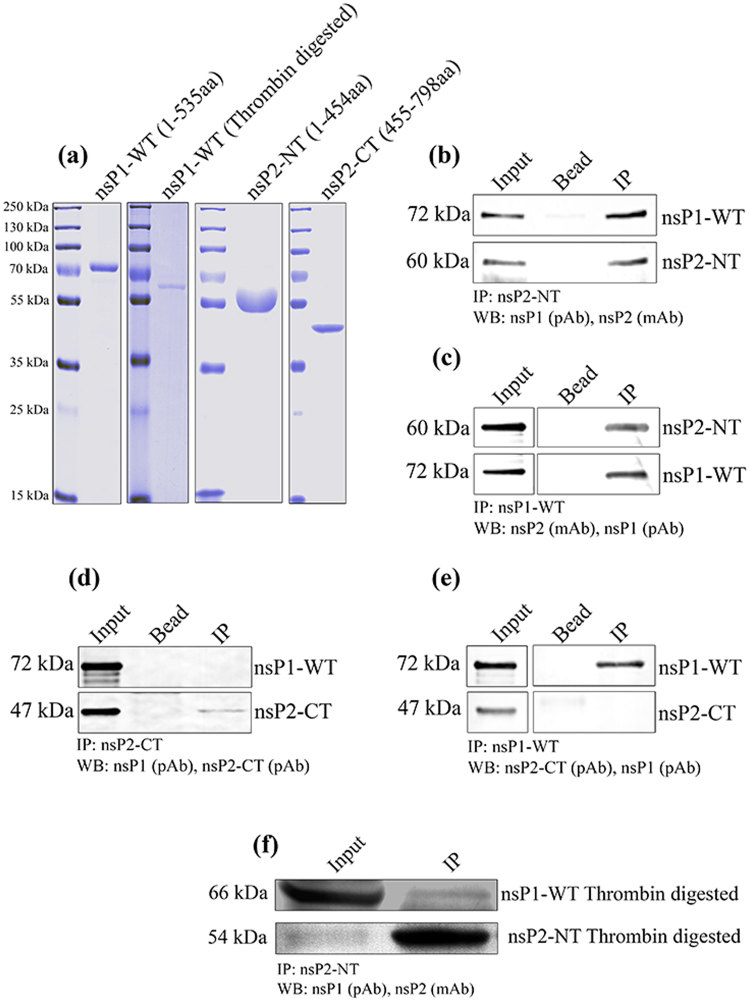
CHIKV nsP2-NT directly interacts with nsP1-WT *in vitro*. (**a**) Coomassie stained 12% SDS-PAGE showing recombinant nsP1-WT, thrombin digested nsP1-WT, nsP2-NT and nsP2-CT. Purified recombinant nsP1-WT and nsP2-NT were incubated for interaction *in vitro* and were either immunoprecipitated with anti-nsP2 mAb (**b**) or anti-nsP1 pAb (**c**) and the same protein or the respective interacting partners were detected by the Western blot. The CHIKV nsP2-CT was incubated with nsP1-WT *in vitro*, the protein complexes were immunoprecipitated with either nsP2-CT pAb (**d**) or nsP1 pAb (**e**) and detection was carried out with specific antibodies in the Western blot. (**f**) Thrombine digested and purified nsP1-WT and nsP2-NT were subjected to IP using anti-nsP2 mAb and Western blot was carried out using anti-nsP1 pAb and anti-nsP2 mAb.

To establish the interaction between CHIKV nsP2 and nsP1 *in vitro*, bacterial expressed His-tagged recombinant proteins were used for co-immunoprecipitation experiments. The CHIKV full-length nsP2 is around 90 kDa protein and contains a cytotoxic property^38^ which makes it difficult to express in the bacterial system. To overcome this problem, N (NT = 1-454 aa) and C (CT = 455-798 aa) terminal parts of nsP2 were expressed separately for this study. Both CHIKV nsP1-WT and nsP2 (NT and CT) were cloned into the pBiEx-1 vector, over-expressed and purified. The pBiEx-1 vector has NT-His-tag and added an extra 6 kDa mass to all the above proteins. The purity of these proteins was around 95%, which was observed in coomassie stained SDS-PAGE (Fig. 2a). A single band was observed for nsP1-WT in the position of around 72 kDa (Fig. 2a). The additional 6 kDa protein was cleaved by thrombin and a 66 kDa band was observed which is shown in the next panel. The protein sequence of nsP1-WT was confirmed by mass spectrometry analysis (Table S6). However, for nsP2-NT, along with the 60 kDa band which is the actual nsP2-NT, other lower size bands (50, 43, 40, 30 kDa) were detected (data not shown). These small fragments were always present in the purified fraction in spite of the extensive washing with higher amount of imidazole with increasing concentration of protease inhibitor. Further, mass spectrometry and Western blot with anti-nsP2 mAb identified the four lower size bands as degraded products of nsP2-NT (Table S6). Finally, the purified fractions containing the 60 kDa protein was subjected to size exclusion chromatograpgy, purified and showed more than 98% purity (Fig. 2a), however, the protein is not very stable. Similarly, nsP2-CT was purified and a single band was observed (47 kDa) as shown in Fig. 2a. To perform the *in vitro* binding assay, an equal concentration of the purified CHIKV nsP1-WT and nsP2-NT or -CT were incubated and different parameters like temperature, timing of interaction, concentration of the proteins and buffer conditions were standardized for optimum binding. Here, nsP2-NT (60 kDa) showed an interaction with CHIKV nsP1-WT (72 kDa) when immunoprecipitated with either anti-nsP2 mAb or anti-nsP1 pAb (Fig. 2b and c). In contrast, no binding was observed for nsP2-CT and nsP1-WT (Fig. 2d and e). The bead and antibody which was used as negative control did not show any binding. Further, to rule out that, the 6 kDa additional protein is not involved in this interaction; the extra part was removed by thrombin digestion and passed through the Ni column. The thrombin digested and purified nsP1-WT and nsP2-NT proteins were subjected to IP. It was observed that these two proteins were interacting without the extra 6 kDa part (Fig. 2f). Hence, the results indicate that, CHIKV nsP2-NT (1-454aa) but not nsP2-CT (455-798aa) interacts directly with nsP1-WT without any mammalian or viral proteins.

### The residues between 170 to 288 aa of nsP1 is responsible for the interaction with nsP2-NT

**Figure 3:**
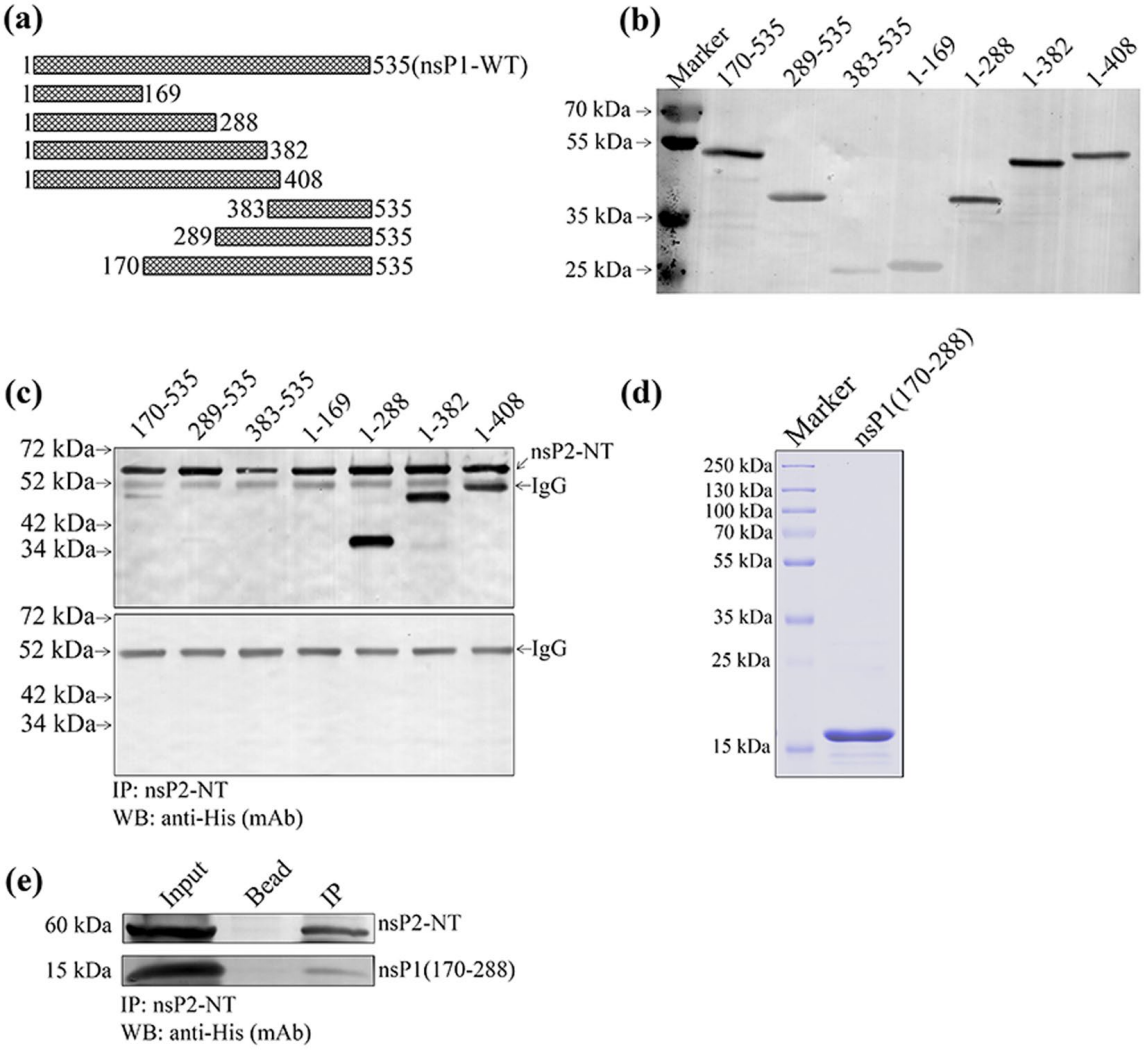
The residues between 170 to 288 aa of nsP1 is responsible for the interaction with nsP2-NT. (**a**) Graphical representation of different truncations of CHIKV nsP1 indicating specific amino acid positions. (**b**) The CHIKV nsP1 truncations were over-expressed in BL-21 cells. The Western blot showing the different CHIKV nsP1 truncated proteins using anti-His mAb. (**c**) Both the CHIKV nsP1 truncated and nsP2-NT proteins were incubated at 4 °C for 2 hr for interaction *in vitro*. The protein complexes were immunoprecipitated using anti-nsP2 mAb, separated in 12% SDS-PAGE and the Western blot was probed with anti-His mAb. The lower panel shows the negative control where different nsP1 trancated proteins were immunoprecipitated with nsP2 mAb and beads. (**d**) Coomassie stained 12% SDS-PAGE showing the purified fragment of nsP1 (170-288). (**e**) The 170-288 aa long purified fragment of nsP1 was incubated with purified nsP2-NT. The protein complex was immunoprecipitated with anti-nsP2 mAb and the Western blot was probed with anti-His mAb. The bead (with nsP2 mAb) was considered as negative control.

To map the region of CHIKV nsP1 responsible for binding with nsP2-NT, different fragments were generated in the region of NT, CT and MB domains of nsP1 (Fig. 3a). All the fragments of CHIKV nsP1 were expressed in the BL-21 cell at 18 °C and analyzed by Western blot which shows that the seven truncated proteins were in the supernatant fraction of the lysed bacterial cells and the His tag was intact for all of them (Fig. 3b). Equal amounts of nsP1 truncated proteins (soluble fraction) were incubated with nsP2-NT at 4 °C for 2 hr and the complex was immunoprecipitated using anti-nsP2 mAb and Western blot were performed using anti-His mAb. A 60 kDa band of nsP2-NT was detected along with the nsP1 truncations 1-288, 1-382, 1-408 (Fig. 3c). Whereas, a faint band was observed for nsP1 truncation 170-535 and the other fragment 289-535 was almost undetectable (Fig. 3c). On the other hand, no band was observed in case of the nsP1 truncations 1-169 and 383-535 (Fig. 3c). The data have been summarized in Table 1, which indicate that the region between 170 to 288 aa (119 aa residues) of nsP1 might be responsible for the binding with nsP2-NT. Next, to find out whether this 119 aa long fragment of nsP1 can be sufficient to keep the interaction with nsP2-NT, the small fragment was purified (Fig. 3d) and IP was performed with nsP2-NT (purified). It was observed that the small fragment (170-288 aa) was immunoprecipitated with nsP2-NT, however, the intensity of the band was less in comparison to the fragments 1-288, 1-382 and 1-408 (Fig. 3e). This observation suggests that 170 to 288 aa position of nsP1 is capable of maintaining the interaction with nsP2-NT; however, the region between 1-169 aa might have some role to make this interaction stronger.

**Table 1:** Analysis of the binding abilities of nsP1 truncations with the nsP2-NT protein.

Truncation name	Amino acid Position	nsP2-NT binding activity (+/−)
nsP1-WT	1-535	+++
nsP1(1-169)	1-169	−
nsP1(1-288)	1-288	+++
nsP1(1-382)	1-382	+++
nsP1(1-408)	1-408	+++
nsP1(383-535)	383-535	−
nsP1(289-535)	289-535	+/−
nsP1(170-535)	170-535	++

(+++): Strong interaction, (++): Moderate interaction, (+): Poor interaction, (−): No interaction.

### The 1-95 aa position of CHIKV nsP2-NT is responsible for interaction with nsP1

**Figure 4:**
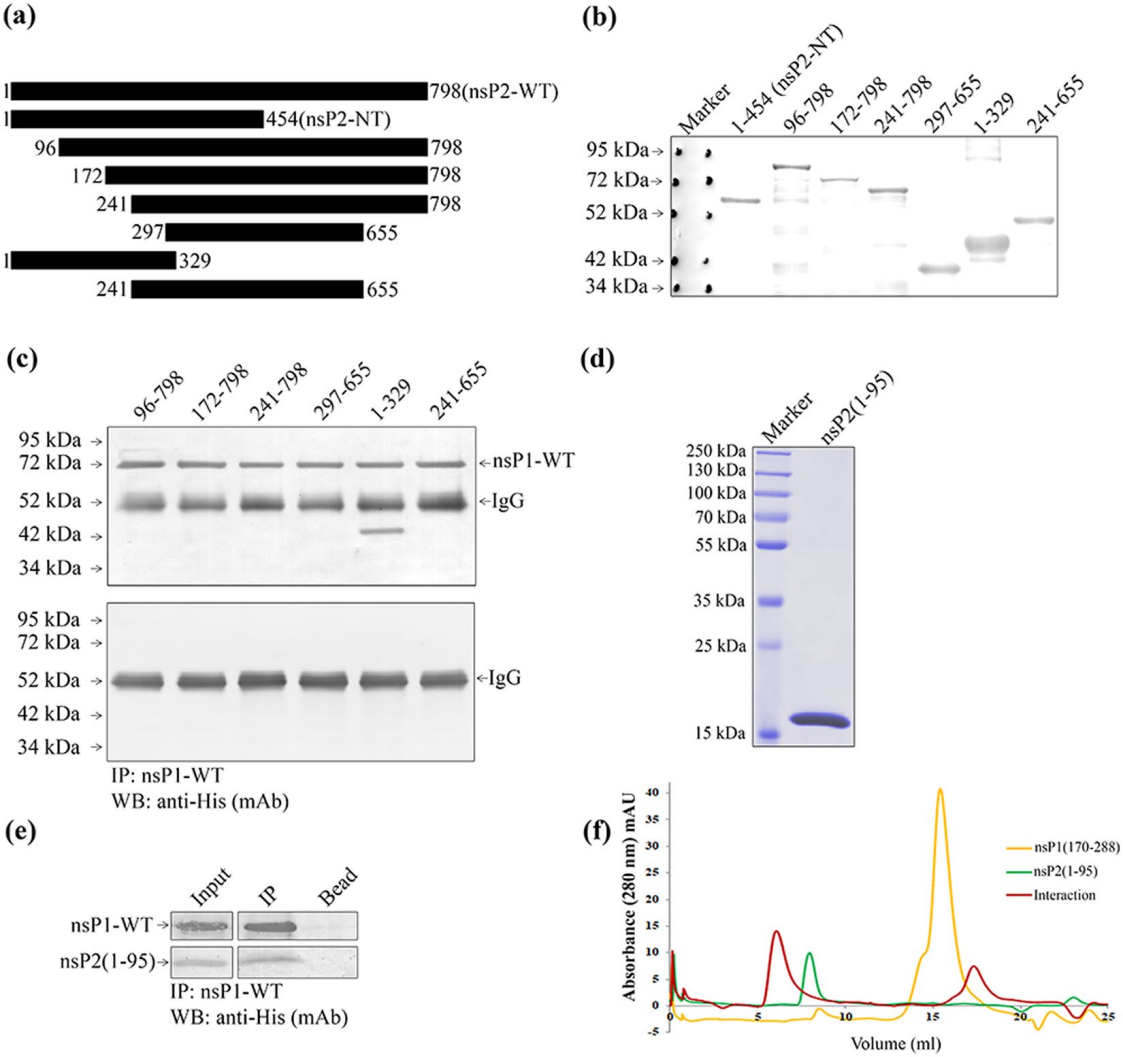
The residues between 1-95 aa of CHIKV nsP2-NT is responsible for the interaction with nsP1-WT. (**a**) Graphical representation of different truncations of CHIKV nsP2 indicating specific amino acid positions. (**b**) The CHIKV nsP2 truncated proteins were over-expressed in BL-21 cells. The Western blot showing the CHIKV nsP2 truncated proteins using anti-His mAb. (**c**) Both the CHIKV nsP2 truncated and nsP1-WT proteins were incubated at 4^o^ C for 2 hr *in vitro* for interaction. The protein complexes were immunoprecipitated using anti-nsP1 pAb and separated in 12% SDS-PAGE. The Western blot was probed with anti-His mAb. The lower panel shows the negative control where different nsP2 trancated proteins were immunoprecipitated with nsP1 pAb and beads. (**d**) Coomassie stained 12% SDS-PAGE showing the purified 1-95 aa fragment of nsP2. (**e**) The purified 1-95 aa long fragment of nsP2 was incubated with purified nsP1-WT. The protein complex was immunoprecipitated with anti-nsP1 pAb and the Western blot was probed with anti-His mAb. The bead (with nsP1 pAb) was considered as negative control. (**f**) Analytical size exclusion chromatography was performed using Superdex 200PG 10/300 column with nsP1 (170-288) and nsP2 (1-95) fragments. Chromatogram showing the eluted volume of nsP1 (170-288) (yellow), nsP2 (1-95) (green) and interacted proteins (red) in 280 nm absorbance.

In order to identify the region of nsP2-NT (1-454aa) responsible for binding with nsP1, truncations were constructed in nsP2-NT as shown in Fig. 4a. The truncated nsP2 proteins were over-expressed and Western blot was performed using the anti-His mAb (Fig. 4b). The over-expressed CHIKV nsP1-WT lysate was incubated with different nsP2-NT truncations, the immune-complex was immunoprecipitated using anti-nsP1 pAb and Western blot showed that only one fragment 1-329 aa of nsP2 was able to interact with nsP1-WT (Fig. 4c). While, the other fragments (96-798, 172-798, 241-798, 297-655 and 241-655) did not show any interaction with nsP1-WT. Taken together, the data indicate that the 1-95 aa long region of nsP2-NT might be responsible for the binding with nsP1-WT (Table 2). Next, the 1-95 aa long fragment of nsP2 was expressed and purified (Fig. 4d) and immunoprecipitated with purified nsP1-WT. In Western blot the small fragment was detected with nsP1-WT when immunoprecipitation was performed using nsP1 pAb (Fig. 4e). The above data suggests that the region 1-95 of nsP2 is capable to interact with nsP1-WT. Further, to confirm the interactin between the two fragments of nsP1 and nsP2, gel filtration chromatography was performed using the purified proteins. It was observed that, the nsP1 (170-288) was eluted at 15.52 ml column volume, nsP2 (1-95) was eluted at 8.16 ml column volume and the complex of the two proteins was eluted at 5.56 ml column volume (Fig. 4f). A shift of approximately 2.6 ml from the elution volume of nsP1 (1-95) indicates strong interaction between both the protein fragments. The above data suggest that, the 1-95aa residues of nsP2 and 170-288 residues of nsP1 are enough to maintain the interaction.

**Table 2:** Analysis of the binding abilities of nsP2 truncations with the nsP1-WT protein.

Truncation name	Amino acid Position	nsP1-WT binding activity (+/−)
nsP2-NT	1-454	+++
nsP2-CT	455-798	−
nsP2(96-798)	96-798	−
nsP2(172-798)	172-798	−
nsP2(241-798)	241-798	−
nsP2(297-655)	297-655	−
nsP2(1-329)	1-329	+++
nsP2(241-655)	241-655	−

(+++): Strong interaction, (++): Moderate interaction, (+): Poor interaction, (−): No interaction.

### Protein-protein docking analysis shows the specific amino acids responsible for the nsP1-nsP2 interaction

**Figure 5:**
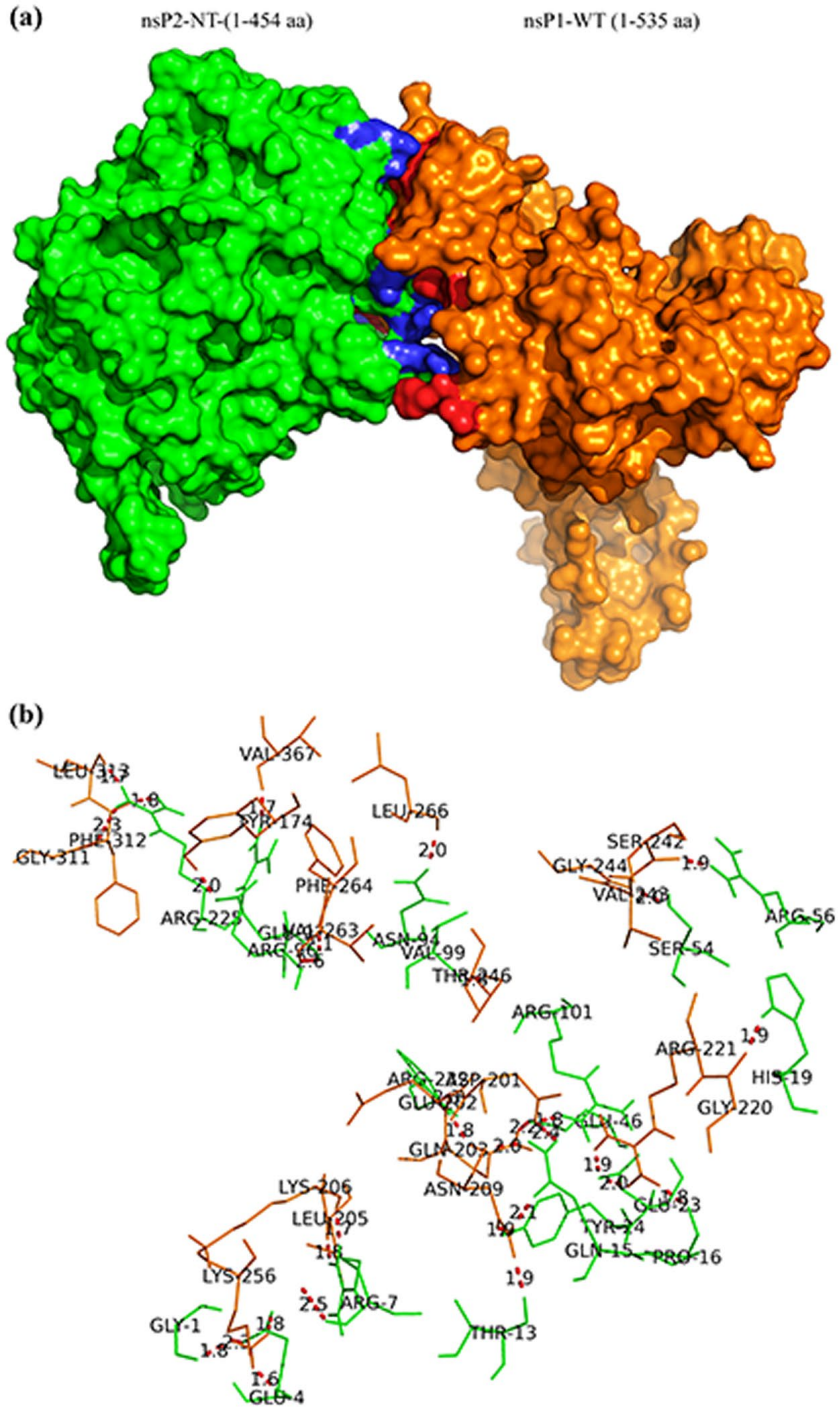
Protein-protein docking analysis shows the specific amino acids responsible for the nsP1-nsP2 interaction. The protein-protein docking between nsP1-WT and nsP2-NT was performed using the ClusPro 2.0 web server. (**a**) The most stable conformation of the complex of nsP2-NT (green) and nsP1-WT (orange). Polar interacting residues within 2.6 Å distance of nsP1-WT (blue) and nsP2-NT (red) are marked in the complex. (**b**) The red bridges showing the polar interactions between residues of nsP1-WT (Orange) and nsP2-NT (Green).

To support the above experimental findings, protein-protein docking was carried out using homology models of nsP2-NT and nsP1-WT. From the results generated by this experiment, the balanced outputs were preferred, as this mode takes into account all possible modes of interaction^39^. The cluster populations are suggested to be proportional to cluster probability^39^. Accordingly, the docking solution with highest (128) number of members were selected for analysis. For this docking solution, a score of −1834.6 KJ/mol was observed for the centre of cluster, while the lowest energy structure was found with a score of −1927.8 KJ/mol (Table S7). The centre of the largest cluster structure rather than the lowest energy structure is generated by the ClusPro, as it accounts for some entropic effects^40,41^. Accordingly, the centre of the largest cluster structure was used for further analysis. Visualization using the PyMol software showed interaction involving the residues observed in the earlier experiment (Fig. 5a). Several polar interactions were also observed among the residues of nsP1 and nsP2 (Table S1). In agreement with experimental findings, fifteen polar interactions were observed within 2 Å of nsP2-NT (1-95 aa) and nsP1 (170-288 aa). Some of these include the interaction of Gly-1, Glu- 4, Arg-7, Thr-13, Pro-16, His-19, Glu-23, Tyr-24, Glu-46, Ser-54, Arg-56 and Asn-94 of nsP2-NT with the residues of nsP1 at Lys-256, Lys-256, Lys-206, Asn-209, Arg-221, Gly-220, Arg-221, Asn-209, Gln-203, Gly-244, Val-243 and Leu-266 respectively (Fig. 5b).

### CHIKV nsP1 affects the ATPase activity of nsP2-NT *in vitro*

**Figure 6:**
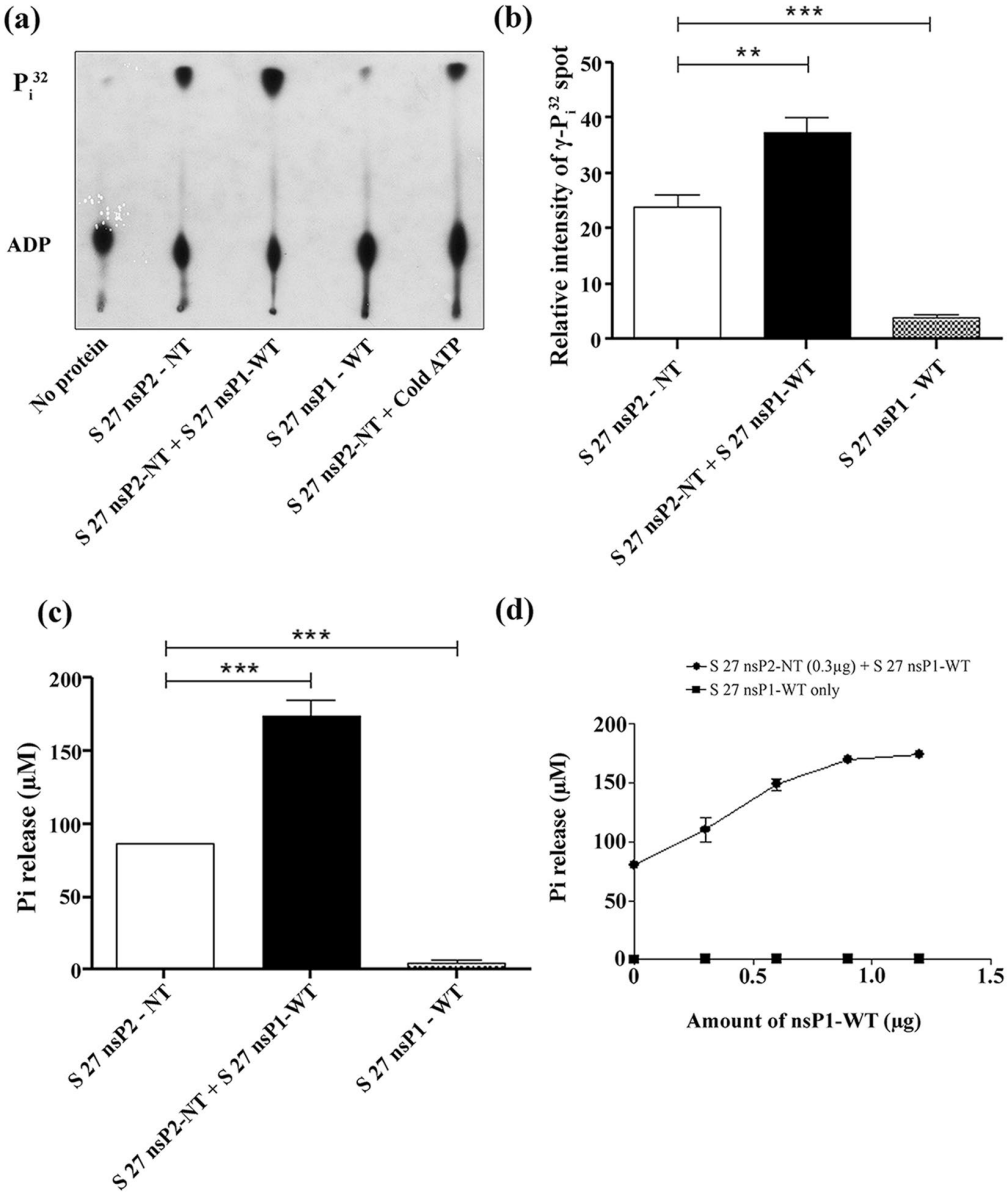
CHIKV nsP1 modulates the ATPase activity of nsP2-NT *in vitro*. (**a**) The ATPase activity was assessed for CHIKV S 27 nsP2-NT and nsP1-WT. The TLC plate was exposed to the X-Ray film and the image was developed. The unlabelled ATP (cold ATP) was used as competitor. (**b**) Bar diagram showing the relative intensities of free γ-P^32^ spots that were released by S 27 nsP1-WT/nsP2-NT alone or together. (**c**) The bar diagram represents the ATPase activities of nsP1-WT or nsP2-NT alone or together which were measured by colorimetric assay. (**d**) The line diagram showing the ATPase activities of nsP1-WT or nsP2-NT with increasing concentrations of nsP1-WT as measured by colorimetric assay. The data represent mean ± SEM of at least three independent experiments. The p-value ≤ 0.05 was considered as statistically significant difference between the groups. (*P ≤ 0.05).

To assess the ATPase activity of the nsP2-NT (1-454 aa) fragment, ATPase assay was performed as mentioned in methods with increasing concentration of this enzyme (0–4 μg). It was observed that the release of free Pi was increasing with the increased amount of nsP2-NT, which was detected by malachite green colorimetric assay (data not shown). As nsP1-WT binds to the nsP2-NT, it was hypothesized that there might be some effect of nsP1 on the ATPase activity of nsP2. Hence, an ATPase assay (TLC assay) was performed either with nsP2-NT (0.2 μg) or with nsP2-NT + nsP1-WT (0.6 μg). It was observed that the release of the free Pi was increased by 1.5 fold in the presence of nsP1-WT (Fig. 6a and b). In addition, a colorimetric assay was also performed. It was noticed that at a concentration of 1 μg of nsP2-NT the Pi release was approximately 86 μM, however, in the presence of 1 μg of nsP1-WT this value was increased to approximately 174 μM (2 fold) (Fig. 6c). Further, it was observed that, with the increasing concentration of nsP1-WT (0-1.2 μg), the release of the free Pi was also enhanced with 0.3 μg of nsP2-NT (Fig. 6d). The ATPase activity of nsP1-WT was also measured and it was negligible in comparison to nsP2-NT (Fig. 6a,b,c and d). Together, the data suggest that the ATPase activity of nsP2-NT was significantly modulated in the presence of nsP1-WT protein *in vitro*.

## Discussion

CHIKV is a mosquito-borne virus, which has created an alarming threat in the modern world due to unavailability of vaccine and antiviral drugs. Moreover, in recent times several outbreaks have been reported in Europe, Asia and America^42,43^. This emphasizes the need to understand the biology of this virus for developing control strategies against this disease. In this study, we showed that CHIKV nsP2 co-localizes as well as interacts with nsP1 during infection in Vero cells through RC. Moreover, it was demonstrated *in vitro* that this interaction is direct and the residues 1-95 aa of nsP2 and 170-288 aa of nsP1 play a major role in maintaining this interaction. Further, an enhancement of CHIKV nsP2 ATPase activity was observed in the presence of nsP1.

Previously, two studies were reported on interaction among CHIKV nsPs where it was shown that, the nsP1 MT and MB domains bind to nsP2 helicase domain^13,36^. The above findings were verified by Y2H, ELISA and GST pull-down assay using the over-expressed bacterial cell lysates. However, no functional relevance of this interaction was shown during CHIKV replication. In this investigation, the CHIKV nsP1-nsP2 interaction was demonstrated in Vero cell which was infected with CHIKV strains. RIPA buffer was used here for lysing the cells and washing the complex of IP. This is a stringent buffer which is often used to rule out any weak and non-specific interaction. Hence, the present observation indicates that the interaction between nsP1-nsP2 was strong. Further, a direct interaction was demonstrated between CHIKV nsP2-NT and nsP1-WT using bacterial expressed purified proteins, suggesting that the two proteins contain the residues which are self-sufficient to maintain the interaction. This interaction was also demonstrated for S 27 and DRDE-06 strains of CHIKV which suggests that, it might not be a strain specific event and might have a vital role in viral replication. Earlier, it was reported that the CHIKV nsP3 protein interacts with nsP1 but not with nsP2^13,36^, however, the current study did not show the presence of nsP3 in the nsP1-nsP2 complex during infection. This might be because of the low level of nsP3 expression^44^ and/or its degradation during infection^45^. Previous reports stated that, the nsP4 protein of SFV directly binds to nsP1 protein in the RC^20^ and during this study, it was also demonstrated that CHIKV nsP4 is present in the nsP1-nsP2 complex which needs further investigation.

Previously, two fragments (167-630^22^ and 1-470^21^) of nsP2 were reported to be expressed stably and purified. However, in this investigation nsP2-NT fragment (1-454 aa) was observed to be degraded partially even though all the precautions were taken. This might be an indication of structural instability of the nsP2-NT fragment without the CT part. Earlier studies demonstrated that, the 167-630 residues of CHIKV nsP2 contains NTPase and RNA tri-phosphatase activities^22^ and further, it was also reported that nsP2 possesses helicase property; however, along with the NT, part of the protease domain (470-798 aa) has been found to be essential for this activity^21^. Though, 1-167 aa residues of CHIKV nsP2 might form a stable functional domain, no important function was predicted to be associated with it^21^. In this study, it was observed that the 1-95 aa of nsP2 was responsible and sufficient enough to maintain the interaction with nsP1. Further investigation can be carried out to identify the important residues in nsP2-NT involved in the nsP1-nsP2 interaction.

Earlier, there was a report in Dengue virus that, the ATPase/helicase activity of NS3 was being modulated due to the interaction with NS5^46^. Likewise, there was a report in Hepatitis C virus, where researchers have shown that an interaction between NS3 and NS4A enhances the ATPase activity which finally helps in the helicase activity of the NS3 protein^47^. For CHIKV nsP2 the conserved ATP binding motifs (Walker A and Walker B) are mapped between aa residues 167-630^22^. In this investigation, it was noticed that the ATPase activity of CHIKV nsP2-NT is enhanced in the presence of nsP1 protein which does not contain ATPase activity. Hence, it can be speculated that the 1-95 residues of nsP2 which is outside the ATPase domain might have a regulatory function for its ATPase function. The binding of nsP2 with nsP1 might lead to conformational changes of nsP2-NT which in turn can enhance/modulate its ATPase activity. The ATPase activity is an important requirement for nsP2 helicase function^21,48^, therefore an increase in ATPase activity might modulate the helicase function of nsP2 which is crucial for CHIKV replication. Future experimental supports are needed to validate the above speculation.

CHIKV nsP1 is a membrane binding protein, which helps in the attachment of RC to the plasma membrane during viral replication^49^. The 171-300 aa region of CHIKV nsP1 has been predicted to contain MB property through bioinformatics analysis^13^ and the amphipathic peptides (245-264 aa) are responsible for this activity^49^. During this study the 170-288 aa region was demonstrated to be essential for binding with nsP2 which indicates that this region might not be involved in membrane binding only and needs further experimental evidences to map the domains precisely. In order to understand whether lack of interaction of truncated proteins are not because of protein misfolding, two non-interacting truncated proteins (289-535 of nsP1 and 455-798 of nsP2) were purified and circular dichroism (CD) was performed. It was observed that they were folded properly (Fig. S1). Accordingly, it can be concluded that, the protein misfolding was not responsible for the lack of interaction. Further, detailed investigation is essential to identify the important residues related to these different functions of nsP1.

The nsP1 protein which is a RNA capping enzyme play a major role in protecting the viral RNA from cytoplasmic ribonucleases as the host capping enzymes are restricted to the nucleus only^50,51^. Previous report on SFV stated that, the RNA triphosphatase activity of alphavirus nsP2 is required for carrying out the capping activity of nsP1^52^. Moreover, for transfer of the 7-methyl-GMP to the viral RNA, the capping apparatus may bind to the RNA genome. However, no RNA binding motif has been reported for the nsP1 protein. Accordingly, from the current observation, it can be speculated that CHIKV nsP1-nsP2 interaction might be useful in RNA capping function of nsP1.

Protein-protein docking analysis suggested the polar interacting residues (nsP1 and nsP2-NT) in the most stable conformation of the complex. Additionally, the sequence similarity analysis (1-95 of nsP2 and 170-288 of nsP1) in 36 different strains of CHIKV showed that, most of the residues are conserved indicating their significance in the interaction (Tables S2 and S3). Strong polar interactions based on low H-bond length (≤1.7 Å) are predicted between LYS-256, LYS-206, VAL-367, and PHE-312 for nsP1 and GLU-4, ARG-7, ARG-96 and ARG-225 for nsP2 respectively (Table S1). In future, mutational study can be designed to explore their role in CHIKV nsP1-nsP2 interaction. In conclusion, these findings have provided support to characterize the interaction between CHIKV nsP1 and nsP2 which might be useful for rational designing of antiviral drugs against this virus.

## Methods

### Cells, viruses and antibodies

Vero cells (African green monkey kidney cell line, a kind gift from Dr. M. M. Parida, DRDE, Gwalior) were maintained according to the procedure described earlier^37^. Both the CHIKV strains, S27 and DRDE-06 (GenBank accession number AF369024.2 and EF210157.2) were the generous gifts from Dr. M.M. Parida. The primary antibodies for nsP2 (mAb)^53^, nsP1 and nsP4 (pAbs)^44^ and nsP2-CT (pAb)^37^ were developed by us and used in this study. Anti-His mAb (αM) (Sigma) was used for Western blot analysis. In addition, the alkaline phosphatase conjugated secondary antibodies, anti-mouse (αM) and anti-rabbit (αR) IgGs (Promega) were used in this study^44^. The normal αM and αR IgGs (Abgenex) were used in IP experiments. The Alexa Fluor 488 and 594 goat αR and αM secondary antibodies (Life Technologies) were used for immunofluorescence assay.

### CHIKV infection

CHIKV infection was carried out in Vero cells as described earlier in a bio safety level-2 (BSL-2) facility at the Institute of life Sciences, Bhubaneswar, India^37^. The 80% confluent cells were infected with either S 27 or DRDE-06 strain of CHIKV at MOI 2. The Cytopathic effect was observed under microscope at a 20× optical magnification and the infected cells along with mock were harvested at different time points according to the need of experiment.

### SDS-PAGE and Western blot

SDS-PAGE and Western blot analyses were performed according to the procedure described earlier^37^. In general, proteins were separated on 10% or  12% SDS-PAGE and transferred onto a nitrocellulose membrane (GE Healthcare). The transferred proteins were probed with anti-CHIKV-nsP2 (mAb), anti-CHIKV-nsP1 (pAb), anti-CHIKV-nsP2-CT (pAb) and anti-His (mAb) followed by secondary antibodies. The blots were developed by using NBT/BCIP reagent (Promega) and scanned by using GS-800 Calibrated Densitometer (Bio-Rad).

### Immunoprecipitation

IP protocol was followed as described previously with minor changes^54^. In brief, approximately 7 × 10^6^ Vero cells were infected with either S 27 or DRDE-06 at MOI 2. The cells were harvested at 10 hpi for S 27 and 6 hpi for DRDE-06 strain and lysed with RIPA buffer. The supernatants were collected, pre-cleared and subjected to IP using protein G-conjugated agarose beads according to the manufacturer’s instructions (GE Healthcare). After that, the beads were washed five times with RIPA buffer and the bound proteins were analyzed in Western blot.

### Immunofluorescence assay (IFA)

The cells, grown on a cover slip (Himedia), were infected with CHIKV and processed for IFA as described earlier^53^. The cells were incubated with anti-CHIKV-nsP1 (pAb) and anti-CHIKV-nsP2 (mAb) antibodies with dilutions 1:125 and 1:500 respectively, followed by secondary antibodies, Alexa Fluor 488 goat anti-rabbit IgG (1:1000) in 3% BSA or Alexa Fluor 594 goat anti-mouse IgG (1:750) in 1× PBS. The cells were stained with 4, 6-diamidino-2-phenylindole (DAPI; Life technology) and mounted in ProLong Gold Antifade mounting media (Life technology). The images were acquired by using the Leica TCS SP5 Confocal microscope (Leica Microsystems) at 63× objective and analyzed by the Leica Application Suite Advanced Fluorescence (LASAF) V.1.8.1 software.

### Cloning, expression and purification

The protein sequences of nsP1 and nsP2 of CHIKV prototype strain, S 27 were retrieved from GenBank accession No: AAN05101.1. For optimum expression in the bacterial system, the genes were codon optimized and synthesized (GenScript, USA). The genes were sub-cloned into the pBiEx-1 vector which has NT-His tag (adding around 6 kDa fragment at the N-terminal end of each clone) by using primers with BamHI and XhoI restriction sites (Tables S4 and S5). Different truncations of CHIKV nsP1 and nsP2 were generated on the basis of the predicted structures^44,55^. The DNA sequences of the clones were confirmed by automated DNA sequencer (Applied biosystem 3500 series genetic analyzer). The plasmids were transformed into BL-21 (DE3) E. coli cells (Stratagene) for protein expression. The previously described protocol was followed for purification^56^. In brief, the transformed BL-21 cells were grown in Luria Broth (LB; HiMedia) medium with 100 µg/ml of ampicillin (Sigma) at 37 °C over night, 180 rpm and was used as a starter culture which was inoculated further for exprsssion with the same previous conditions. Once the optical density (OD) reached at 0.6-0.8, 0.3 mM isopropyl-D-1-thiogalactopyranoside (IPTG; Sigma) was added and induced at 18 °C for 16 hr, 180 rpm. The cell pellets were collected by centrifugation at 8,000 rpm for 8 min at 4 °C and re-suspended in Buffer A (50 mM Tris-HCl pH 7.5, 500 mM NaCl, 10 mM imidazole, 10% glycerol, 1 mM β-mercaptoethanol with protease inhibitor cocktail (Roche). The cells were disrupted by sonication, using a Digital Sonifier 450 (Branson) at 40% amplitude for 5-10 min or by French press. The lysed sample was clarified by centrifuging for 2 h at 17,500 × g and 4 °C. The supernatant of the clarified sample was collected and passed through a His-trap FF Nickel affinity column (GE healthcare) pre-equilibrated with buffer A and purified by stepwise gradient using Buffer A and Buffer B (20 mM Tris-HCl pH7.5, 300 mM NaCl, 250 mM imidazole, 1 mM β-mercaptoethanol and 1 mM PMSF). The eluted protein peak fractions were concentrated to a volume of 5.0 ml and chromatographed over a HiLoad 16/600 Superdex 200 prep grade (PG) column (GE Healthcare) with buffer C (20 mM Tris-HCl pH7.5, 300 mM NaCl, 1 mM β-mercaptoethanol and 1 mM PMSF) at a flow rate of 0.5 ml/min on AKTA Pure M machine (GE Healthcare) in the cold. Fractions containing pure recombinant protein were collected, combined and stored at −80 °C. The protein content and purity were evaluated after every stage of purification with SDS-PAGE and Coomassie Brilliant Blue R-250 stained gels.

### *In vitro* immunoprecipitation assay

The purified recombinant nsP1-WT was incubated with nsP2-NT/CT for 2 hrs at 4 °C. After this, 10 μg of anti-nsP1 pAb/ anti-nsP2 mAb/ anti-nsP2-CT pAb was added and incubated for 2 hrs. To precipitate the protein complexes, 30 μl of G-beads were added and incubated overnight. The same amount of protein was incubated with specific antibodies and beads, which serve as a negative control. The IP complex was washed 10 times with RIPA buffer followed by washing with 1× PBS. The proteins were eluted by heating at 100 °C for 5 min in 1× SDS lysis buffer with PBS and protease inhibitors. To map the amino acids for nsP1-nsP2 interaction, the over-expressed truncations were lysed using bacterial lysis buffer with lysozyme (100 mg/ml), freeze-thawed 5 times and incubated with either nsP1-WT or nsP2-NT proteins as per the experimental requirements. Then, the above mentioned IP and Western blot procedure were carried out using desired antibodies.

### Analytical Size Exclusion Chromatography (SEC)

Analytical SEC was performed for nsP1 (residues 170-288) and nsP2 (residues 1-95) using Superdex 200 10/300 GL column equilibrated with buffer containing 20 mM Tris-HCl pH7.5, 150 mM NaCl, 1 mM β-mercaptoethanol and 1 mM PMSF. 80 µM of both nsP1 (residues 170-288) and nsP2 (residues 1-95) proteins were chromatographed on the column separately. For interaction study, 24 µM concentration each of both nsP1 (residues 170-288) and nsP2 (residues 1-95) were mixed together in a total of 500 µl volume, incubated on ice for 30 minutes and chromatographed on the column.

### Protein-protein docking studies

The protein-protein docking between nsP1 and nsP2-NT was performed using the ClusPro 2.0 webserver^39,57^. This server performs three computational steps. The first step involves rigid-body docking using PIPER, a docking program based on the Fast Fourier Transform (FFT) correlation approach that uses pair wise interaction potential as part of its scoring function E = w_1_E_rep_ + w_2_E_attr_ + w_3_E_elec_ + w_4_E_DARS._ E_rep_ and E_attr_ represent the repulsive and attractive contributions to the van der Waals interaction energy. E_elec_ denotes electrostatic energy term and E_DARS_ refers to the pair wise structure-based potential^39,58^. The 1000 lowest energy docked structures are clustered using pair wise interface RMSD (IRMSD) as the distance measure in the second step^39,59^. The IRMSD values for each pair among these structures are calculated to determine the structure with highest neighbors within a 9 Å radius. This is defined as the centre of the first cluster. This is then removed and similar clustering performed within the remaining structures to generate 30 clusters. In the third step the energy minimization is done for the structures using the van der Waals terms of the CHARMM potential^39,60^. Following this the structures at the centre of the ten most populated clusters are taken as the output.

The output generated four types of models using the scoring algorithms designated as balanced, electrostatic-favored, hydrophobic favored and van der waals + electrostatic. Due to unavailability of crystal structures of both the proteins, their homologous models were used as the input structure. Since there was no satisfactory template available in PDB to build the homologous models, the structures were generated earlier using the I-TASSER algorithm and reported^44,55^. The CHIKV nsP2-NT was taken as the receptor and nsP1-WT was used as the ligand. Acidic and basic residues of interacting regions (1-95 aa of nsP2-NT and 170-288 aa of nsP1) of both the proteins which are most likely to participate in the polar interaction were selected as attractive residues before submission of the job. The output generated four types of models using the scoring algorithms designated as balanced, electrostatic-favored, hydrophobic-favored and van der waals + electrostatic. Amongst these, the balanced outputs were analyzed. The docking solution with largest (128) members (Table S7) was taken for further visualization using the PyMol software.

### ATPase assay (Colorimetric)

The ATPase assay was performed by measuring phosphate release using a colorimetric method based on complex formation with malachite green and molybdate as described earlier with little modifications^22,61^. Briefly, in a 50 µl reaction, 1× ATPase reaction buffer [50 mM HEPES (pH 7.6), 2 mM MgCl_2_, 10 mM KCl, 0.05 mg of bovine serum albumin (BSA) per ml, 2 mM DTT], 1 mM ATP and enzyme were added. The reaction was performed at 37 °C for 30 min and terminated by EDTA (20 mM). Finally an O.D was measured at 630 nM.

### ATPase assay (Thin Layer Chromatography, TLC)

The ATPase assay^22,48^ was carried out in 20 µl reaction volume containing 1 × ATPase reaction buffer, 0.5 µCi of [γ-P^32^] ATP (3000 Ci/mmol, BRIT, India). The mixture was incubated at 37 °C for 30 min. To observe the free γ-P^32^, 1 µl of reaction mixture was spotted onto a polyethyleneimine-cellulose TLC plate (Merck) and separated by using 0.375 M Potassium phosphate (pH 3.48) as the mobile phase. The plate was air dried and exposed to X-ray film. The spot intensity was quantified by using the Image J software.

### Statistical Analysis

The statistical analysis was performed by using the One-way ANOVA method (nonparametric and Dunnett’s Multiple Comparison Test) in Graph Pad Prism 5.0 software and the data were presented as mean ± SEM of three independent experiments (n ≥ 3). The p-value less than equal to 0.05 was considered as significant for all the statistical analysis.

## Electronic supplementary material

Supplementary Information
