# Supplementary material for: Chikungunya virus nsP1 interacts directly with nsP2 and modulates its ATPase activity
Source: Sci Rep. 2018 Jan 18;8:1045. doi: 10.1038/s41598-018-19295-0 (PMC5773547; doi:10.1038/s41598-018-19295-0)

Chikungunya virus nsP1 interacts directly with nsP2 and modulates its ATPase activity.

Sameer Kumar[1], Abhishek Kumar[1], Prabhudutta Mamidi[1], Atul Tiwari[2], Sriram Kumar[3], Animamalar Mayavannan[3], Sagarika Mudulli[1], Ajit Kumar Singh[1], Bharat Bhusan Subudhi[4], Soma Chattopadhyay[1*].

[1]Institute of Life Sciences, Bhubaneswar, India

[2] Banaras Hindu University,Varanasi,U.P., India

[3]Anna University, Chennai, India

[4]School of Pharmaceutical Sciences, Siksha O Anusandhan University, Bhubaneswar, India

[*]**Address of Corresponding author:**

Soma Chattopadhyay

Institute of Life Sciences,

Autonomous Institute of Dept of Biotechnology (Govt of India),

Nalco Square, Bhubaneswar-751023, India

Phone No: 0091 674 2301676; Fax No: 0091 674 2300728

Email: sochat.ils@gmail.com

Key Words: Chikungunya, nsP1, nsP2, Alphavirus, ATPase

Supplementary Table S1. Interacting residues of nsP1-WT and nsP2-NT from docking analysis.

Sl No.	nsP1 (Orange)	nsP2-NT (Green)	H-bond (Å)
1	LYS-256	GLY-1	1.8
2	LYS-256	GLU-4	1.6
3	LYS-256	GLU-4	2.3
4	LEU-255	ARG-7	2.5
5	LEU-205	ARG-7	1.8
6	LYS-206	GLU-4	1.8
7	LYS-206	ARG-7	1.7
8	ASN-209	THR-13	1.9
9	ASN-209	GLN-15	2.1
10	ASN-209	TYR-24	1.9
11	GLN-203	GLU-46	2.2
12	GLN-203	ARG-232	1.8
13	GLU-202	ARG-232	2.3
14	ASP-201	ARG-101	1.8
15	ASP-201	GLN-15	2.4
16	ARG-221	GLU-23	1.9
17	ARG-221	GLU-23	2.0
18	ARG-221	PRO-16	1.8
19	GLY-220	HIS-19	1.9
20	THR-246	VAL-99	1.8
21	VAL-243	ARG-56	1.9
22	SER-242	ARG-56	1.9
23	GLY-244	SER-54	2.0
24	VAL-263	GLU-97	2.6
25	VAL-263	GLU-97	2.1
26	PHE-264	GLU-97	2.1
27	LEU-266	ASN-94	2.0
28	TYR-174	GLU-97	2.0
29	VAL-367	ARG-96	1.7
30	GLY-311	ARG-225	2.3
31	PHE-312	ARG-225	1.7
32	LEU-313	ARG-225	1.8

Red coloured residues are deviations from experimental observations. H-bond ≤ 2 Å can be taken as cut off to show polar interaction.

Supplementary Table S2. Homology analysis of nsP2 (1-95 aa) with 36 different strains* of CHIKV.

Strain	Sequence (1-95)
CHIKV nsP2(1-95)	GIIETPRGAIKVTAQPTDHVVGEYLVLSPQTVLRSQKLSLIHALAEQVKTCTHSGRAGRYAVEAYDGRVLVPSGYAISPEDFQSLSESATMVYNE
AAU43880.1 Senegal_37997_1983	....I..........................
ABD95937.2 LR2006 OPY1 (Re-Union)	....N...
ABJ98543.1 D570/06 (Mauritius)	....N...
ABN04187.1 IND-06-AP3	....N...
ABN04189.1 IND-06-KA15	....N...
ABN04191.1 IND-06-MH2	....N...
ABN04193.1 IND-06-RJ1	....N...
ABN04195.1 India_TN1_2006	....N...
ABN04197.1 IND-00-MH4 (Yawat)	....
ABN04201.1 India_MH5_1973	...............L...
ABP88821.1 DRDE-06	....N...
ABU93704.1 Wuerzburg (Mauritius)	....S..N...
ABX38963.1 ITA07-RA1 (Long Varient)	....N...
ACD93567.1 MY002IMR/06/BP	...............L...
ACD93607.1 TM25 (Mauritius)	....N...
ACD93609.1 CHIK31 (Rajsthan)	....N...
ACY25937.1 KL06, RGCB03	....N...
ACY25939.1 KL06	....N...
ACY25943.1 KL07	....N...
ACY25947.1 KL08 INDIA 2008	....N...
ACY66840.1 Singapore_0611aTw_2006	....N...
ACY66844.1 Bangladesh_0810aTw_2008	....N...
ACY66846.1 Malaysia_0810bTw_2008	....N...
ADG95884.1 Nigeria_IbH35_1964	....I..........................
ADG95905.1 Indonesia_RSU1_1985	...............L...
ADG95912.1 Sri-lanka_CK1_2007	....N...
ADG95927.1 Congo_LSFS_1960	....
ADG95929.1 Thailand_TH35_1958	...............L...
ADG95931.1 Tanzania_Ross low-psg_1953	....
ADG95933.1 Uganda_Ag4155_1982	....
ADG95935.1 India_Gibbs263_1963	...............L...
ADG95937.1 Thailand_1455_1975	...............L...
ADJ19189.1 Thailand_CU10_2008	....N...
ADJ19191.1 Thailand_CU683_2009	....N...
BAH97930.1 Japan_SL11131_2006	....N...
CAJ90474.1 La Reunion_06-021_2005	....N...

*nsPs protein sequences of different CHIKV strains from all over the world isolated at different period of times which was retrieved from PubMed.

Supplementary Table S3. Homology analysis of nsP1 (170-288 aa) 36 different strains* of CHIKV.

nsP1(167-288)	V R L A Y W V G F D T T P F M Y N A M A G A Y P S Y S T N W A D E Q V L K A K N I G L C S T D L T E G R R G K L S I M R G K K L E P C D R V L F S V G S T L Y P E S R K L L K S W H L P S V F H L K G K L S F T C R C D T V V S C E G Y V V K
AAU43880.1 Senegal_37997_1983	. . V . . . I . . . . M K . . . .
ABD95937.2 LR2006 OPY1 (Re-Union)	. . V . . . . K . . . .
ABJ98543.1 D570/06 (Mauritius)	. . V . . . . K . . . .
ABN04187.1 IND-06-AP3	. . V . . . . K . . . .
ABN04189.1 IND-06-KA15	. . V . . . . K . . . .
ABN04191.1 IND-06-MH2	. . V . . . . K . . . .
ABN04193.1 IND-06-RJ1	. . V . . . . K . . . .
ABN04195.1 India_TN1_2006	. . V . . . . K . . . .
ABN04197.1 IND-00-MH4 (Yawat)	. . V . . . . K . . . .
ABN04201.1 India_MH5_1973	. . V . . . . K . . . . T . . . .
ABP88821.1 DRDE-06	. . V . . . . K . . . .
ABU93704.1 Wuerzburg (Mauritius)	. . V . . . . K . . . .
ABX38963.1 ITA07-RA1 (Long Varient)	. . V . . . . R . . . K . . . .
ACD93567.1 MY002IMR/06/BP	. . V . . . . K . . . . M . . . .
ACD93607.1 TM25 (Mauritius)	. . V . . . . K . . . .
ACD93609.1 CHIK31 (Rajsthan)	. . V . . . . K . . . .
ACY25937.1 KL06, RGCB03	. . V . . . . K . . . .
ACY25939.1 KL06	. . V . . . . K . . . .
ACY25943.1 KL07	. . V . . . . K . . . .
ACY25947.1 KL08 INDIA 2008	. . V . . . . K . . . . R . . . .
ACY66840.1 Singapore_0611aTw_2006	. . V . . . . K . . . .
ACY66844.1 Bangladesh_0810aTw_2008	. . V . . . . T . . . . R . . . K . . . .
ACY66846.1 Malaysia_0810bTw_2008	. . V . . . . K . . . .
ADG95884.1 Nigeria_IbH35_1964	. . V . . . I . . . . M . . . .
ADG95905.1 Indonesia_RSU1_1985	. . V . . . . K . . . . M . . . .
ADG95912.1 Sri-lanka_CK1_2007	. Q V . . . . K . . . .
ADG95927.1 Congo_LSFS_1960	. . V . . . .
ADG95929.1 Thailand_TH35_1958	. . V . . . . L . . . . K . . . . T . . . .
ADG95931.1 Tanzania_Ross low-psg_1953	. . V . . . . K . . . . T . . . .
ADG95933.1 Uganda_Ag4155_1982	. . V . . . . K . . . .
ADG95935.1 India_Gibbs263_1963	. . V . . . . K . . . . T . . . .
ADG95937.1 Thailand_1455_1975	. . V . . . . K . . . . T . . . .
ADJ19189.1 Thailand_CU10_2008	. . V . . . . D . . . . K . . . .
ADJ19191.1 Thailand_CU683_2009	. . V . . . . K . . . .
BAH97930.1 Japan_SL11131_2006	. Q V . . . . K . . . .
CAJ90474.1 La Reunion_06-021_2005	. . V . . . . K . . . .

*nsPs protein sequences of different CHIKV strains from all over the world isolated at different period of times which was retrieved from PubMed.

Supplementary Table S4. Lists of the primers used for generating CHIKV nsP1 truncations.

Primer	Nucleotide sequences (5'–3')[a]	Nucleotide position[b]	Region of nsP1 to amplify (aa)[c]	Pair of Primers used
nsP1-WT-F	TAA**GGATCC**AGACCCGGTGTATGTG	4-18	1-535	nsP1-WT-F and nsP1-WT-R
nsP1-169-R	TAA**CTCGAG**TTAGCCTTTGATCGCTTG	493-507	1-169	nsP1-WT-F and nsP1-169-R
sP1-288-R	TAA**CTCGAG**TTATTTCACGACATAACC	850-864	1-288	nsP1-WT-F and nsP1-288-R
nsP1-382-R	CAG**CTCGAG**TTAGTAGTTTTTCATGGT	1132-1146	1-382	nsP1-WT-F and nsP1-382-R
nsP1-408-R	TAA**CTCGAG**TTACAGCAGTTTTTCGTC	1210-1224	1-408	nsP1-WT-F and nsP1-408-R
nsP1-WT-R	TAA**CTCGAG**TTAAGCACCTGCGCG	1594-1605	1-535	nsP1-WT-R and nsP1-WT-F
nsP1-170-F	TAA**GGATCC**AGTGCGCCTGGCC	508-519	170-535	nsP1-170-F and nsP1-WT-R
nsP1-289-F	TAA**GGATCC**ACGCATTACCATGTCA	865-879	289-535	nsP1-289-F and nsP1-WT-R
nsP1-383-F	TAA**GGATCC**AATGATCCCGGTGGTT	1147-1161	383-535	nsP1-383-F and nsP1-WT-R

[a]- Bold alphabets indicate the restriction enzyme site incorporated to clone in pBiEx-1 vector.
[b] - Nucleotide positions are, according to the optimized CHIKV nsP1 gene sequence.
[c]- Amino acid positions are, according to the sequence of CHIKV S 27 nsP1 protein retrieved from GenBank.

Supplementary Table S5. Lists of the primers used for generating CHIKV nsP2 truncations.

Primer	Nucleotide sequence (5'–3')[a]	Nucleotide position[b]	Region of nsP2 to amplify (aa)[c]	Pair of Primers used
nsP2-NT-F	TAA**GGATCC**AGGCATCATCGAAACC	1-15	1-454	nsP2-NT-F and nsP2-NT-R
nsP2-NT-R	CGA**CTCGAG**TTAATGTTCCACTTCCCA	1348-1362	1-454	nsP2-NT-R and nsP2-NT-F
nsP2-329-R	CGA**CTCGAG**TTATTTACCTTCATAGTG	973-987	1-329	nsP2-NT-F and nsP2-330-R
nsP2-95-R	CGA**CTCGAG**TTATTCATTGTACACCAT	271-285	1-95	nsP2-NT-F and nsP2-95-R
nsP2-CT-F	CGA**GGATCC**AGCTTCGATTATGGC	1363-1376	455-798	nsP2-CT-F and nsP2-CT-R
nsP2-CT-R	TAA**CTCGAG**TTAGCAACCTGCGCG	2383-2394	455-798	nsP2-CT-R and nsP2-CT-F
nsP2-96-F	CGA**GGATCC**ACGCGAATTTGTGAA	286-299	96-798	nsP2-97-F and nsP2-CT-R
nsP2-172-F	TAA**GGATCC**ACGCCCGGCTTGC	514-525	172-798	nsP2-173-F and nsP2-CT-R
nsP2-241-F	TAA**GGATCC**AGGTTGCAACCGTCC	721-734	241-798	nsP2-242-F and nsP2-CT-R
nsP2-297-F	CGA**GGATCC**AAACCATAACATTTGC	889-903	297-655	nsP2-298-F and nsP2-656-R
nsP2-655-R	TAA**CTCGAG**TTACGCAACCCACGTAA	1952-1965	241-655	nsP2-242-F and nsP2-656-R

[a]- Bold alphabets indicate the restriction enzyme site incorporated to clone in pBiEx-1 vector.
[b] - Nucleotide positions are, according to the optimized CHIKV nsP2-NT gene sequence.
[c]- Amino acid positions are, according to the sequence of CHIKV S 27 nsP2 protein retrieved from GenBank.

Supplementary Table S6. Mass spectrometry analysis of nsP1-WT and nsP2-NT bands.

Search title : ILS Proteomics2\ILS-154-09062017\MSMS 8\A2

Database : NCBInrChikv 20160107 (2565 sequences; 1607194 residues

	Spot ID	Best Protein Accession	Best Protein Mass	Best Protein Score	Best Protein Description
nsP1-WT	A7	gi|315435272	60849	55	nonstructural polyprotein, partial [CHIKV]
	A9	gi|16904822	60828	169	nonstructural protein 1, partial [CHIKV]
	A10	gi|325975283	90758	83	nsP2 protein, partial [Chikungunya virus]
nsP2-NT 1st band	A11	gi|325975283	90758	48	nsP2 protein, partial [Chikungunya virus]
	A12	gi|325975283	90758	63	nsP2 protein, partial [Chikungunya virus]
nsP2-NT 2nd band	A15	gi|325975283	90758	76	nsP2 protein, partial [Chikungunya virus]
	A16	gi|325975283	90758	288	nsP2 protein, partial [Chikungunya virus]
nsP2-NT 3rd band	A17	gi|325975283	90758	319	nsP2 protein, partial [Chikungunya virus]
	A18	gi|325975283	90758	426	nsP2 protein, partial [Chikungunya virus]
	A19	gi|325975283	90758	27	nsP2 protein, partial [Chikungunya virus]
nsP2-NT 4th band	A20	gi|325975283	90758	86	nsP2 protein, partial [Chikungunya virus]
	A21	gi|325975283	90758	165	nsP2 protein, partial [Chikungunya virus]
nsP2-NT 5th band	A23	gi|133872768	59792	15	polyprotein, partial [Chikungunya virus]
	A25	gi|16904822	60828	536	nonstructural protein 1, partial [CHIKV]
nsP1 (289-535) band	A26	gi|16904822	60828	249	nonstructural protein 1, partial [CHIKV]
	A27	gi|16904822	60828	423	nonstructural protein 1, partial [CHIKV]

Protein scores greater than 47 are significant (p<0.05).

Search Parameters

Type of search: Sequence Query, **Enzyme**: Trypsin**, Fixed modifications**: Carbamidomethyl (C**), Variable modifications**: Oxidation (M)

Mass values: Monoisotopic, **Protein Mass**: Unrestricted, **Peptide mass tolerance**: ± 200 ppm, **Fragment mass tolerance**: ± 0.8 Da

Max Missed Cleavages: 1, **Instrument type**: MALDI-TOF-TOF

Supplementary Table S7. Population and energy scores of the balanced outputs generated by the ClusPro software.

Cluster	Members	Representative	Weighted Score
0	128	Center	-1834.6
0	128	Lowest Energy	-1927.8
1	57	Center	-1458.3
1	57	Lowest Energy	-1652.2
2	52	Center	-1471.9
2	52	Lowest Energy	-1712.1
3	43	Center	-1451.2
3	43	Lowest Energy	-1780.4
4	41	Center	-1758.1
4	41	Lowest Energy	-1758.1
5	40	Center	-1511.8
5	40	Lowest Energy	-1667
6	38	Center	-1721.2
6	38	Lowest Energy	-1721.2
7	37	Center	-1477.1
7	37	Lowest Energy	-1699.1
8	34	Center	-1562
8	34	Lowest Energy	-1616.2
9	33	Center	-1491.7
9	33	Lowest Energy	-1686.8
10	33	Center	-1522.4
10	33	Lowest Energy	-1526.4
11	32	Center	-1641.6
11	32	Lowest Energy	-1673
12	23	Center	-1651.6
12	23	Lowest Energy	-1704.1
13	23	Center	-1469.9

13	23	Lowest Energy	-1644.7
14	21	Center	-1550.1
14	21	Lowest Energy	-1550.1
15	20	Center	-1497.5
15	20	Lowest Energy	-1640.6
16	19	Center	-1440.3
16	19	Lowest Energy	-1671.9
17	18	Center	-1489
17	18	Lowest Energy	-1661.9
18	18	Center	-1457.2
18	18	Lowest Energy	-1567.6
19	16	Center	-1441.3
19	16	Lowest Energy	-1655.5
20	16	Center	-1513.9
20	16	Lowest Energy	-1560.8
21	16	Center	-1446.1
21	16	Lowest Energy	-1641
22	16	Center	-1482.1
22	16	Lowest Energy	-1599.6
23	15	Center	-1590.7
23	15	Lowest Energy	-1590.7
24	15	Center	-1575.2
24	15	Lowest Energy	-1575.2
25	14	Center	-1454
25	14	Lowest Energy	-1757.3
26	14	Center	-1582.2
26	14	Lowest Energy	-1582.2
27	14	Center	-1542.1
27	14	Lowest Energy	-1542.1
28	14	Center	-1540.1

28	14	Lowest Energy	-1588.4
29	8	Center	-1476.8
29	8	Lowest Energy	-1505.3

Supplementary Figure S1. Original Western blot images of nsP1(left pannel) and nsP2(right pannel) truncations of fig 3b and fig 4b with ladder:

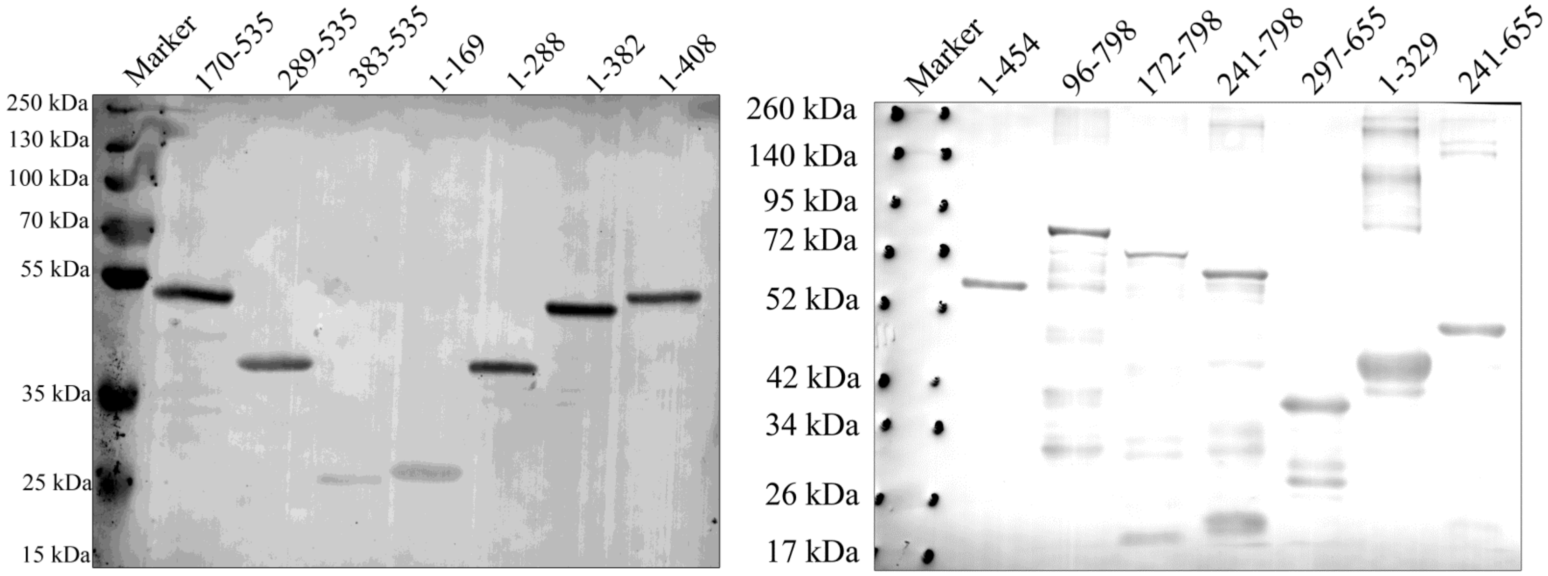

Supplementary Figure S2. Far UV CD spectra of recombinant purified CHIKV non-structural protein fragments: The spectra have been plotted with molar residue ellipticity (MRE) against wavelength and indicate definite secondary structural properties. The measurements were made using a Jasco J-1500 CD spectrophotometer.

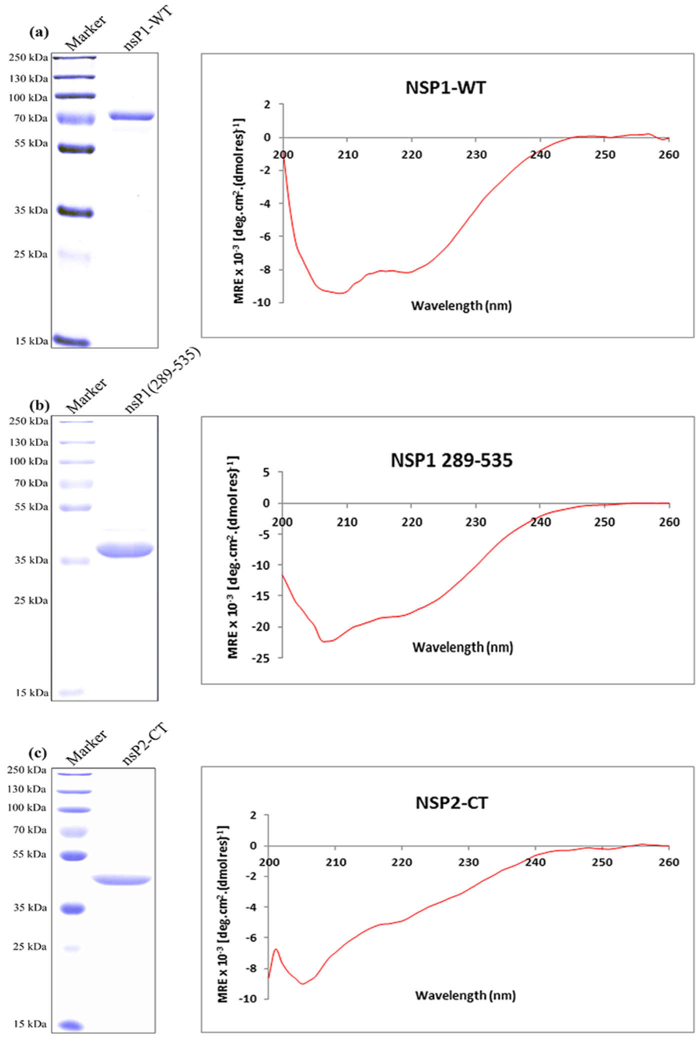

Supplement: Supplementary file 1 — Supplementary Information [file 41598_2018_19295_MOESM1_ESM.pdf]
